# Mini Review: Quantum Confinement of Atomic and Molecular Clusters in Carbon Nanotubes

**DOI:** 10.3389/fchem.2021.796890

**Published:** 2021-12-08

**Authors:** María Pilar de Lara-Castells, Alexander O. Mitrushchenkov

**Affiliations:** ^1^ Instituto de Física Fundamental (AbinitSim Unit), IFF-CSIC, Madrid, Spain; ^2^ MSME, Univ Gustave Eiffel, CNRS UMR 8208, Univ Paris Est Creteil, Marne-la-Vallée, France

**Keywords:** clusters of molecular hydrogen, clusters of atomic helium, carbon nanotubes, quantum confinement, ab initio intermolecular interaction theory, wave-function method for bound-state calculations, full quantum coupled characterizations

## Abstract

We overview our recent developments on a computational approach addressing quantum confinement of light atomic and molecular clusters (made of atomic helium and molecular hydrogen) in carbon nanotubes. We outline a multi-scale first-principles approach, based on density functional theory (DFT)-based symmetry-adapted perturbation theory, allowing an accurate characterization of the dispersion-dominated particle–nanotube interaction. Next, we describe a wave-function-based method, allowing rigorous fully coupled quantum calculations of the pseudo-nuclear bound states. The approach is illustrated by showing the transition from molecular aggregation to quasi-one-dimensional condensed matter systems of molecular deuterium and hydrogen as well as atomic ^4^He, as case studies. Finally, we present a perspective on future-oriented mixed approaches combining, e.g., orbital-free helium density functional theory (He-DFT), machine-learning parameterizations, with wave-function-based descriptions.

## 1 Introduction

The cylindrical confinement provided by carbon nanotubes has offered the possibility of studying the pronounced quantum behaviour of ^4^He atoms and H_2_ molecules at reduced dimensionality. Recent measurements have demonstrated the formation of two-dimensional (2D) ^4^He layers on the outer surface of single-walled carbon nanotubes (SWCNTs) (Noury et al., 2019). Also, an experimental study (Ohba, 2016) of gas adsorption at low (2–5 K) temperature revealed a quenched propagation of ^4^He atoms through carbon nanopores with diameters below 7 Å despite of their small kinetic diameter. The application of orbital-free helium density functional theory (He-DFT) to carbon nanotubes immersed in a helium nanodroplet provided theoretical explication that the experimental observations stem from the exceptionally high zero-point energy of ^4^He as well as its tendency to form two-dimensional (2D) layers upon adsorption at low temperatures (Hauser and de Lara-Castells, 2016). These conclusions were further confirmed by applying more accurate *ab initio* potential modelling along with a wave-function (WF)-based approach (Hauser et al., 2017). This study also showed that SWCNTs are filled by more molecules of N_2_ than ^4^He atoms, due to the higher zero-point energy of the latter. More generally, the interaction of small atomic and molecular systems with carbon-based nanostructures has attracted a lot of attention recently (see, e.g. Deb et al., 2016; Deb et al., 2019; Paul et al., 2019; Paul et al., 2020 and references therein).

**GRAPHICAL ABSTRACT:**
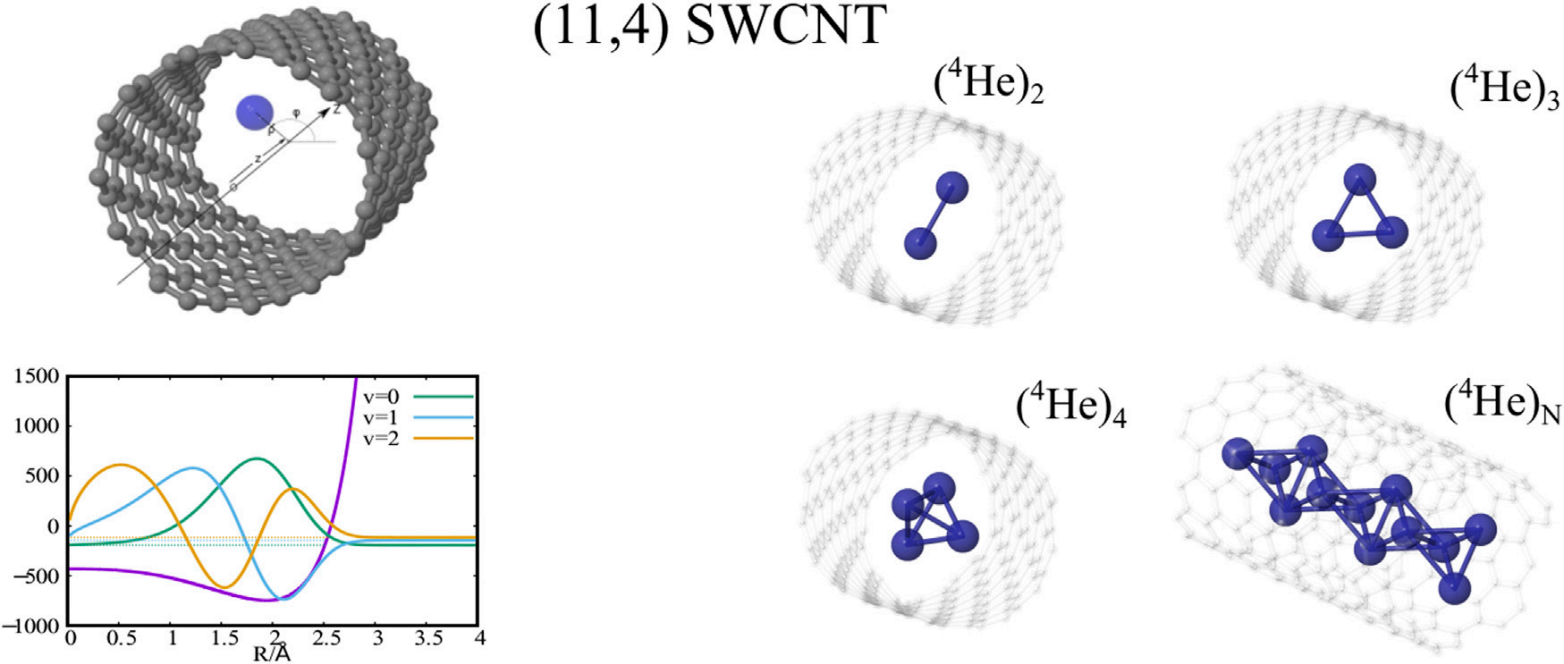


For the case of molecular deuterium, our recent theoretical work has provided conclusive evidence for the transition from molecular aggregation to quantum solid-like packing in a SWCNT of 1 nm diameter (de Lara-Castells and Mitrushchenkov, 2020; de Lara-Castells and Mitrushchenkov, 2021), confirming a previous study using an embedding approach in a broader (ca. 1.4 nm) SWCNT (de Lara-Castells et al., 2017). Experimental evidence, using neutron scattering, on the formation of one-dimensional D_2_ crystals under carbon nanotube confinement has been reported as well (Cabrillo et al., 2021). Altogether, these studies have confirmed the key role played by the quantum nature of the nuclear degrees of freedom in the confined atomic helium, molecular hydrogen, or molecular deuterium motion.

The quest for the understanding of the aggregation of molecular H_2_ in carbon nanotubes is also application-oriented as it is being actively used as a clean energy source, substituting fossil fuels. In fact, its combustion produces only heat and water, and it can be efficiently combined with oxygen in a fuel cell to produce electricity. Yet, the usage of hydrogen as a profit fuel requires a substantial development of efficient storage materials (Schlapbach and Züttel, 2001; Züttel, 2003; Zheng et al., 2021) such as metal organic frameworks, Rosi et al. (2003) covalent organic frameworks (Zheng et al., 2021), and carbon-based nanoporous materials (Cheng et al., 2001; Liu et al., 2010). Hydrogen storage methods are aimed to pack hydrogen molecules as close as possible, existing direct experimental evidences for solid-like packing of hydrogen at temperatures of industrial importance (Ting et al., 2015). Actually, the question on the nature of the hydrogen packing in carbon nanotubes is truly fundamental. Thus, the possible existence of either a superfluid (Rossi and Ancilotto, 2016) or a crystalline phase (Del Maestro and Boninsegni, 2017; Ferré et al., 2017) for para-H_2_ molecules inside carbon nanotubes at zero (Rossi and Ancilotto, 2016; Ferré et al., 2017) or ultra-low temperatures (Del Maestro and Boninsegni, 2017) have been addressed as well (see also (Cazorla and Boronat, 2017) for a recent review). Specially appealing in regards to the impact of quantum effects on the diffusion of H_2_ and D_2_ along carbon nanotubes is the experimental finding of reserved trends in their rates upon cooling (Nguyen et al., 2010), with the D_2_ isotope becoming the faster inspite of its higher mass (Nguyen et al., 2010). The impact of quantum effects in confined H_2_ and D_2_ motion has been theoretically confirmed as well (Mondelo-Martell and Huarte-Larrañaga, 2016; Mondelo-Martell et al., 2017; Mondelo-Martell and Huarte-Larrañaga, 2021).

The high interest on the confinement of clusters of atomic ^4^He is also related to the superfluid nature of ^4^He droplets at a temperature close to absolute zero (0.4 K) (Mudrich and Stienkemeier, 2014) and, more specifically, to the onset of such fascinating property in doped clusters made of just a few ^4^He atoms (Toennies and Vilesov, 2004). Then, the question is whether the confinement provided by SWCNTs can favour the emergence of such feature at reduced dimensionality. The effect of carbon nanotube confinement has been also shown to be remarkable for the ^3^He isotope inside a SWCNT of 1 nm diameter. Thus, as opposed to the free dimer case, the confined ^3^He dimer is predicted to be bound, mainly as a consequence of the strong localization along the radial ^3^He–SWCNT direction (de Lara-Castells and Mitrushchenkov, 2021).

This overview article is organized as follows. The next section presents the computational approach addressing the modeling of intermolecular adsorbate–SWCNT interactions as well as fully coupled quantum calculations of the corresponding pseudo-nuclear wavefunctions. Section 3 presents an illustrative application of the computational approach to the transition from molecular aggregation to quasi-one-dimensional (1D) matter systems composed by either D_2_/H_2_ molecules or ^4^He atoms. Finally, Section 4 closes with a summary of concluding remarks and a few future prospects.

## 2 Computational Approach

### 2.1 *Ab Initio* Modelling of the Adsorbate-Nanotube Interaction

Achieving a correct description of the interaction of the lightest atomic and molecular clusters in nature (i.e., made of He or H_2_) with its confining SWCNT environment is a challenge even for expensive *ab initio* methods since they are dominated by long-range dispersion forces (van der Waals forces). Extended dispersion-corrected DFT methods are applicable including confinement effects in extended SWCNT structures but they bear a tendency to overshoot when applied to ultimate cases of dispersion-dominated (e.g., He-surface) interactions. One key idea has consisted in designing a functional [the so-called dlDF functional (Pernal et al., 2009)] which accounts for the dispersionless interaction energy only so that the dispersion corrections can be safely added later. Within a practical implementation of this idea, the incremental method (Stoll, 1992) is applied on non-periodic (small) cluster models of extended systems and combined with periodic dispersionless DFT calculations (de Lara-Castells et al., 2014b; de Lara-Castells et al., 2014a). This approach has been shown to be particularly successful when describing the interaction of atomic helium (de Lara-Castells et al., 2014a) as well as heavier noble gases (de Lara-Castells et al., 2015) with coronene/graphene/graphite surfaces. Very recently, it has been also demonstrated how modern *ab initio* intermolecular perturbation theory allows a cost-effective and accurate characterization of van der Waals-dominated He-SWCNT and H_2_-SWCNT interactions using small clusters models of the SWCNTs. The use of small cluster models is justified as complicated, dispersionless contributions are mostly of short-range nature. Dispersion, on the other hand, is long-range, but the corresponding parameters show excellent transferability properties upon increasing the size of the surface cluster models (de Lara-Castells et al., 2014a; de Lara-Castells et al., 2015). Therefore, these parameters can be calculated at high level of *ab initio* theory on small clusters and then scaled to the actual SWCNT system. As previously emphasized (de Lara-Castells and Hauser, 2020), detailed energy decomposition schemes, an intrinsic feature of methods such as density functional theory (DFT)-based symmetry-adapted perturbation theory [SAPT(DFT)] (Hesselmann and Jansen, 2003; Misquitta et al., 2003; Heßelmann et al., 2005; Misquitta et al., 2005) has been shown to be particularly useful in this respect.

In our most recent works (de Lara-Castells and Mitrushchenkov, 2020; de Lara-Castells and Mitrushchenkov, 2021), the SAPT(DFT) method has been used to derive by fitting the dispersionless and dispersion SAPT(DFT) energy contributions to an additive pairwise potential model (PPM) (de Lara-Castells et al., 2016; Hauser and de Lara-Castells, 2017; de Lara-Castells et al., 2017; Hauser et al., 2017), which is a modified version of that proposed by Carlos and Cole (Carlos and Cole, 1980). For the illustrative cases presented in this work, these terms were calculated for the interaction between a single H_2_ molecule (He atom) and a hydrogen-saturated (unsaturated) nanotube SWCNT(5, 5) tube made of 62 (40) atoms, considering two transverse sections of the tube. The SAPT(DFT) MOLPRO (Heßelmann et al., 2005; Werner et al., 2012) implementation was applied, using the Perdew-Burke-Ernzerhof (PBE) density functional (Perdew et al., 1996) and the augmented polarized correlation-consistent triple-zeta basis (Woon et al., 1994) for all atoms but the hydrogen atoms saturating the dangling bonds, for which the correlation-consistent double-zeta basis was used instead.

The PPM functional form (de Lara-Castells et al., 2016; Hauser and de Lara-Castells, 2017; de Lara-Castells et al., 2017; Hauser et al., 2017) for the dispersionless energy contribution accounts for the typical exponential growth of the dominant dispersionless term, the exchange-repulsion, but also including a Gaussian-type “cushion” to describe weakly attractive tails stemming from other dispersionless terms

$$E_{\text{int}}^{\text{disp}-\text{less}}\left(\left\{R_{\text{A}-\text{C}}\right\}\right)=\underset{\text{C}}{\sum }\left[1+\gamma_{R}\left(1-\frac{6}{5}\cos^{2}\theta_{\text{C}}\right)\right]\times A\;e^{\left(-\alpha \;R_{\text{A}-\text{C}}-\beta \;R_{\text{A}-\text{C}}^{2}\right)},\;\;\;\;R_{\text{A}-\text{C}}<R_{c},$$ (1)

where *R*_*c*_ is a cut-off distance, *R*_A-C_ stands for the distance between the adsorbate center-of-mass and one carbon atom of the SWCNT, and *θ*_C_ is the angle between the radial vector going from the SWCNT center to one carbon atom and the vector **R**_A-C_ pointing from the adsorbate center-of-mass to the same C atom. The dimensionless factor *γ*_R_ in the first term accounts for the anisotropy of the C − C bonds. The sum in Eq. (1) runs over all carbon atoms of the nanotube. For the dispersion part, we apply the typical C_6_/C_8_ expansion with the damping functions of Tang and Toennies *f*_*n*_ (*n* = 6, 8) (Tang and Toennies, 1984)

$$E_{\text{int}}^{\text{disp}}\left(\left\{R_{\text{A}-\text{C}}\right\}\right)=-\underset{\text{C}}{\sum }\left[1+\gamma_{A}\left(1-\frac{3}{2}\cos^{2}\theta_{\text{C}}\right)\right]\times \underset{n=6,8}{\sum }\frac{\sqrt{C_{n}^{\text{A}}C_{n}^{\text{C}}}}{R_{\text{A}-\text{C}}^{n}}f_{n}\left(\sqrt{\beta_{\text{A}}\beta_{\text{C}}}R_{\text{AC}}\right),$$ (2)

where *γ*_A_ is also a dimensionless anisotropy parameter. The inclusion of *γ*_*A*_ and *γ*_*R*_ anisotropy terms has been found to be important when modelling potential corrugation effects on both curved carbon and metallic surfaces (de Lara-Castells et al., 2016; Hauser and de Lara-Castells, 2017; Hauser et al., 2017).

### 2.2 The Pseudo-Nuclear Wave-Function Problem

**FIGURE 1:**
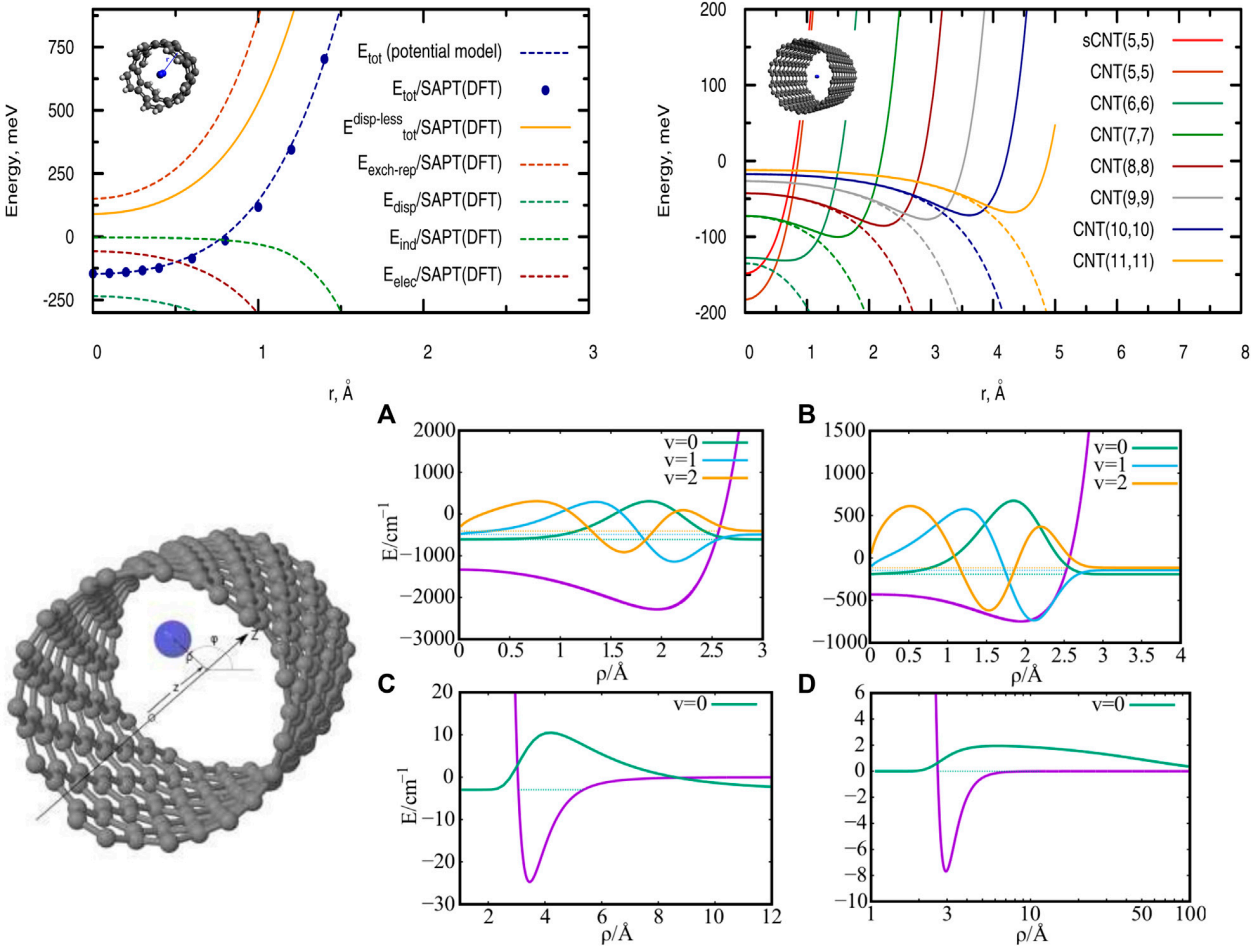
Top panel. Left-hand side: Radial scan of the interaction energies between a single H_2_ molecule and a short (single-walled) carbon nanotube (sCNT) of helicity index (5, 5). The pairwise potential model is compared with reference *ab initio* calculations using the SAPT(DFT) approach. Right-hand side: Radial scan of the total interaction energies (full lines) and dispersion contributions (dashed lines) between a single H_2_ molecule and carbon nanotubes with helicity indexes of increasing value. Source: (de Lara-Castells et al., 2017) Bottom panel. Left-hand side: Cylindrical coordinates describing a single particle in a tube. Right-hand side: interaction potentials PN-CNT **(A, B)** and PN-PN **(C, D)** together with the corresponding low-lying bound states of a single confined PN. **(A, C)**: PN = H_2_, **(B, D)**: PN = ^4^He. Source: (de Lara-Castells and Mitrushchenkov, 2021).

Our method has been designed to allow bound-state calculations for *N* identical atoms or molecules (referred to as “pseudo-nuclei”, PNs) inside a SWCNT. Using cylindrical coordinates (*x*_*i*_, *y*_*i*_, *z*_*i*_ → *ρ*_*i*_, *ϕ*_*i*_, *z*_*i*_) for each PN (see left panel of Figure 1), together with a Jacobian transformation of the volume to keep the Hamiltonian “explicitly Hermitian”, $\Psi \to \sqrt{\rho_{1}\rho_{2}\ldots \rho_{N}}\;\Psi$ , the Hamiltonian describing a motion of *N* PN’s takes the form

$${\hat{H}}_{N}=\overset{N}{\underset{i=1}{\sum }}\left\{-\frac{1}{2M}\left[\frac{\partial^{2}}{\partial \rho_{i}^{2}}+\frac{\partial^{2}}{\partial z_{i}^{2}}+\frac{1}{\rho_{i}^{2}}\left(\frac{\partial^{2}}{\partial \phi_{i}^{2}}+\frac{1}{4}\right)\right]+V_{1}\left(\rho_{i}\right)\right\}+\underset{i<j}{\sum }V_{2}\left(r_{ij}\right)$$ (3)

where the distance *r*_*ij*_ is explicitly given by

$$r_{ij}^{2}=\rho_{i}^{2}+\rho_{i}^{2}+{\left(z_{i}-z_{j}\right)}^{2}-2\rho_{i}\rho_{j}\cos\left(\phi_{i}-\phi_{j}\right).$$ (4)

We note that, in order to preserve the cylindrical symmetry of the system, the very small corrugation appearing along the azimuthal degree of freedom *ϕ* is not accounted for. Considering long nanotubes, it is thus assumed that the interaction potential depends only on *ρ*. The atomic structure of the SWCNT, however, is implicitly considered through the SAPT(DFT) calculations used to fit the parameters of our PPM (see Section 3.1). These potentials are used within the Discrete Variable Representation (DVR) approach (Bačic̀ and Light, 1989) upon fitting them to polynomials of *ρ*:

$$V_{1}\left(\rho \right)=\overset{l_{\max}}{\underset{l=0}{\sum }}p_{l}\rho^{l}$$ (5)

To separate the overall *Z*-translation and overall rotational motions, we introduce relative coordinates as *t*_*i*_ = *z*_*i*_ − *z*_*N*_ and *χ*_*i*_ = *ϕ*_*i*_ − *ϕ*_*N*_, for *i* = 1 … *N* − 1. The overall *Z*-translation and overall rotation coordinates are conveniently defined as $Z=Z_{\text{COM}}=\frac{1}{N}\sum_{i=1}^{N}z_{i}$ and *Φ* = *ϕ*_*N*_. The overall rotation quantum number reads *Λ* = *m*_1_ + *m*_2_ + ⋯ + *m*_*N*_, where *m*_*i*_ is the integer standing for the projection of the angular momentum of the *i*-th particle onto the nanotube axis. Since both PN–SWCNT and PN–PN interactions do not depend on *Φ*, the coordinate *Φ* can be separated via the factor $\exp\left(i\Lambda \Phi \right)\Psi \left(\chi_{i}\right)/\sqrt{2\pi }$ . Finally, the full kinetic energy is written as $K_{N}=-\frac{1}{2M}\sum_{i=1}^{N}\Delta \left(r_{i}\right)=K_{N}\left(z\right)+K_{N}\left(\rho \right)+K_{N}\left(\phi \right)$ where

$$K_{N}\left(z\right)=-\frac{1}{M}\overset{N-1}{\underset{i=1}{\sum }}\frac{\partial^{2}}{\partial t_{i}^{2}}-\frac{1}{M}\overset{N-1}{\underset{i<j=1}{\sum }}\frac{\partial }{\partial t_{i}}\frac{\partial }{\partial t_{j}}$$ (6)

$$K_{N}\left(\rho \right)=-\frac{1}{2M}\overset{N}{\underset{i=1}{\sum }}\frac{\partial^{2}}{\partial \rho_{i}^{2}}$$ (7)

and

$$K_{N}\left(\phi \right)=-\frac{1}{2M}\overset{N-1}{\underset{i=1}{\sum }}\frac{1}{\rho_{i}^{2}}\left[\frac{\partial^{2}}{\partial \chi_{i}^{2}}+\frac{1}{4}\right]-\frac{1}{2M}\frac{1}{\rho_{N}^{2}}\left[{\left(i\Lambda -\overset{N-1}{\underset{i=1}{\sum }}\frac{\partial }{\partial \chi_{i}}\right)}^{2}+\frac{1}{4}\right]$$ (8)

We note that the internal Hamiltonian does not account explicitly for the bosonic permutation symmetry of all *N* particles. The symmetry is automatically included for the particles labelled as 1 … *N* − 1 since it is equivalent to a simple exchange of the corresponding coordinates *t*_*i*_, *ρ*_*i*_, *χ*_*i*_. The exchange of the particles labelled as *i* and *N*, however, results in linear transformations of the coordinates *t*_*i*_ and *χ*_*i*_. The symmetry with respect to the *i* ↔ *N* exchange is analyzed *a posteriori* by defining a “bosonic symmetry factor” *Q* as the matrix element:

$$Q=\left\langle \Psi_{i}|\left(1↔N\right)|\Psi_{j}\right\rangle$$ (9)

The *Q* factor is unity for true bosonic solutions. This test is used as a criteria to evaluate the wavefunction accuracy.

To calculate the eigenvalues of the internal Hamiltonian, we use the DVR approach (Bačic̀ and Light, 1989; de Lara-Castells and Mitrushchenkov, 2020; de Lara-Castells and Mitrushchenkov, 2021). This approach allows an easy and quick evaluation of the pair interaction potential *V*_2_, with the basis set being obtained as a direct product of functions for the different coordinates. Sinc-DVR functions are conveniently employed for the *t* and *χ* coordinates. When dealing with the polar radii *ρ*, however, it is necessary to explicitly treat the singular kinetic energy term $-1/8M\rho_{i}^{2}$ , which stems from the Jacobian transformation to cylindrical coordinates. For this purpose, we use the DVR basis obtained for the finite basis set representation (FBR) built from the radial functions of two-dimensional (2D) Harmonic oscillator functions. However, this basis renders the grid size too large for clusters with, e.g., 4 PNs since the internal Hamiltonian becomes 10-dimensional (10 D). This problem is efficiently solved by using potential-optimized DVR (PO-DVRs) functions (Echave and Clary, 1992), allowing for a very fast energy convergence as well. The diagonalization of the resulting Hamiltonian matrix is carried out using the Jacobi-Davidson algorithm (Sleijpen and van der Vorst, 1996). This technique has been very successful even for characterizing ill-behaved interactions such as, e.g., the “hard-core” interaction problem of doped helium clusters (de Lara-Castells et al., 2009a; de Lara-Castells et al., 2009b).

## 3 Illustrative Applications

In the first subsection, we will illustrate how the interaction of molecular hydrogen with various SWCNTs can be modelled at *ab initio* level. In the second subsection, we will show how our computational approach has allowed to reveal the transition from van-der-Waals-type molecular aggregation to quasi-1D condensed matter systems for atomic ^4^He and molecular H_2_ and D_2_ inside a SWCNT of 1 nm diameter.

### 3.1 H_2_-Nanotube Interaction Potentials for SWCNTs of Increasing Diameter

As an illustrative case, the top (left-hand) panel of Figure 1 shows the total H_2_/CNT interaction potential as a function of the radial distance *r* between the molecule center-of-mass and the SWCNT(5,5) center, along with the electrostatic E_elec_, exchange-repulsion E_exch-rep_, induction E_ind_, and dispersion contributions E_disp_. Upon applying the pairwise PPM presented in Section 3.1, the radial scans of interaction energies shown in the upper (right-hand) panel of Figure 1 are obtained for SWCNTs of increasing diameter.

As can be observed in the top panel of Figure 1 (left-hand side), the H_2_/CNT attractive interaction is dispersion-dominated, with the exchange-repulsion dominating the whole dispersionless term. This repulsive energy contribution grows exponentially as the molecule-surface distance decreases although such behaviour is somewhat smoothed out by the attractive electrostatic contribution. The induction term contributes very little at the potential minimum but considerably at the SWCNT cage. The potential minimum is located at the center of the narrowest nanotube (diameter of 6.74 Å), where the adsorbates benefit from the dispersion interaction with carbon atoms at both sides of the carbon cage. However, upon increasing the nanotube diameter (see right-hand panel at the top of Figure 1), the dispersion becomes very small at the nanotube center and the potential minima shift towards the carbon cage. For a nanotube of diameter of 27.21 Å, the value of the well-depth (−56 meV) is already close to that obtained for a graphene sheet and measured for the H_2_ molecule adsorbed onto graphite (−51.7 meV from Ref. 29). From the top (left-hand side) panel of Figure 1 it can also be observed that the pairwise potential model closely reproduces the results obtained with the SAPT(DFT) method. Similar conclusions hold for the case of the He/SWCNT interaction (Hauser et al., 2017), also emphasizing the usefulness of the SAPT(DFT)-derived PPM (see Section 3.1).

### 3.2 From van-der-Waals Aggregation to Quasi-One-Dimensional Chains

**FIGURE 2:**
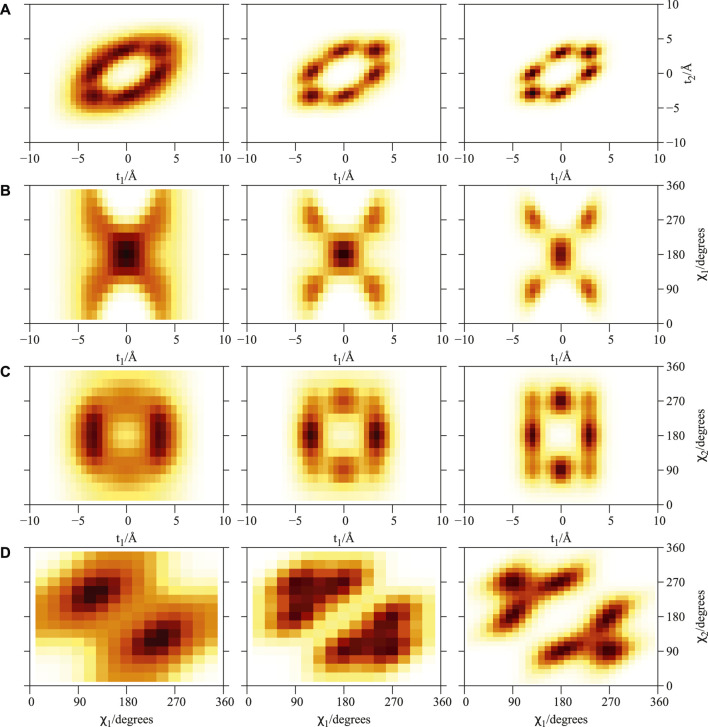
Plot of 2D densities for the ground state of PN_4_ ⊂ SWCNT(11, 4) complexes. Left to right: PN = ^4^He, para-H_2_, ortho-D_2_. **(A)** *t*_1_ and *t*_2_ coordinates; **(B)** *t*_1_ and *χ*_1_ coordinates; **(C)** *t*_1_ and *χ*_2_ coordinates; **(D)** *χ*_1_ and *χ*_2_ coordinates. Source: (de Lara-Castells and Mitrushchenkov, 2021).

Considering a SWCNT of 1 nm diameter and helicity index (11, 4), the He-SWCNT and H_2_-SWCNT one-particle potentials *V*_1_(*ρ*) are shown in the bottom (right-side) panels of Figure 1 along with a few supported bound states of a single ^4^He atom or H_2_ molecule. Figure 2 presents the plots of the ground-state two-dimensional (2D) densities of PN_4_ clusters (PN = ^4^He, para-H_2_, and ortho-D_2_).

As previously reported (de Lara-Castells and Mitrushchenkov, 2020; de Lara-Castells and Mitrushchenkov, 2021) the structuring of PN_*N*_ clusters, with *N* = 1 − 3, is characterized by a high pseudo-nuclear delocalization. Thus, the triangular-like structure exhibited by confined (^4^He)_3_ clusters (see graphical abstract) freely rotates inside the nanotube. However, as can be observed in Figure 2, whatever the bosonic particle be, the most probable structure is pyramidal-like. This confinement effect is specially remarkable for the case of ^4^He, due to the high delocalization of the bare PN–PN dimer (see bottom panel (d) of Figure 1). Yet, it features an increasing spatial delocalization when going from molecular ortho-D_2_ through molecular para-H_2_ to atomic ^4^He. Inspite of para-H_2_ molecules being twice as light as ^4^He atoms, the three times deeper attractive well of the pair potential (see bottom panels (a) and (b) of Figure 1) causes a much more compact structuring of the (para-H_2_)_4_ cluster when compared with the ^4^He counterpart. Particularly, we note that the extended profile of the 2D densities in the *χ* coordinates reflects a quasi-independent relative rotational motion of two (^4^He)_2_ dimers lying orthogonal to the tube axis. Since ortho-D_2_ molecules are twice as heavy as para-H_2_ molecules, the solid-like nature of the (ortho-D_2_)_4_ structure (a regular tetrahedron) becomes even more apparent than for the (para-H_2_)_4_ counterpart. The formation of (vibrationally averaged) tetrahedral structures of (para-H_2_)_4_ clusters is not a special effect from the SWCNT confinement. In fact, the same feature has also been revealed in clathrate hydrate cages (Sebastianelli et al., 2008; Witt et al., 2010). These similarities indicate that the aggregation of multiple light PNs are driven by quantum nuclear effects and very weak dispersive PN–PN interaction forces rather than the much more stronger PN–SWCNT (or PNT-cage) energy contribution (see bottom panels (a) and (b) of Figure 1). As discussed in detail in our previous work (de Lara-Castells and Mitrushchenkov, 2020), the onset of solid-like packing for molecular deuterium is explained by analyzing the potential minima landscape, allowing also to predict the formation of a one-dimensional chain of tetrahedral structures along the tube axis when the number of D_2_ molecules increase. Similar conclusions holds for the cases of ^4^He atoms and H_2_ molecules (de Lara-Castells and Mitrushchenkov, 2021). Moreover, these special structures feature stabilization of collective rotational motion resembling the behaviour of quantum rings exhibiting persistent current (charged particles) or persistent flow (neutral particles). This characteristic has been connected with the onset of superfluid motion and persistent flow in quantum rings made of ^4^He atoms by Bloch (Bloch, 1973). It is remarkable that such feature occurs already in clusters made of four ^4^He atoms at the reduced dimensionality offered by the confining medium.

## 4 Concluding Remarks and Future Directions

Summarizing, this mini review shows how a challenging case of confined molecular system can be accurately characterized using multi-scale first-principles modelling. In order to emphasize the key role of quantum effects, we have chosen the lightest atomic and molecular species in nature as the confined object and SWCNTs as the confining medium. In the first part, we have overviewed how an *ab initio*-derived pairwise potential model can accurately characterize the intermolecular interaction between the confining medium and the confined object. In the second part, we have illustrated how a computational approach, allowing for rigorous fully coupled quantum calculations of the pseudo-nuclear bound states, has been capable of revealing the transition from van der Waals-type molecular aggregation to quasi-1D condensed matter systems under cylindrical carbon nanotube confinement. Remarkably, it has been also shown that the structuring is driven by purely dispersive PN-PN interactions together with quantum pseudo-nuclear effects and not the much more stronger PN-tube interaction forces. In this way, it can be also understood why, inspite of its weak cohesive PN-PN interaction, neutron scattering experimental measurements have very recently shown the formation of quasi-one-dimensional structures of molecular D_2_ corresponding to cylindrical sections of the hexagonal close-packed bulk crystal (Cabrillo et al., 2021).

As a future perspective, we are aimed to adapt our Full Configuration Interaction Nuclear Orbital (FCI-NO) approach (de Lara-Castells et al., 2009b), originally developed for doped helium clusters, to the present case in which the carbon nanotube acts as the “dopant” species. The essential advantage of this *ansatz* is that the wave function is expanded using products of nuclear orbitals, allowing the employment of second quantization techniques, an automatic inclusion of Fermi–Dirac and Bose–Einstein nuclear spin statistical effects, and, then, a characterization of ^3^He and ^4^He isotopes on an equal footing. The second future prospect is the creation of embedding approaches linking our wave-function-based description with orbital free He-DFT (Dalfovo et al., 1995; Ancilotto et al., 2017), following previous efforts (Hauser et al., 2017). In this sense, the highly accurate method presented in our latest work (de Lara-Castells and Mitrushchenkov, 2021) is expected to provide benchmark results guiding new, machine-learning driven, parameterizations in He-DFT, as already illustrated in electronic structure theory (Meyer et al., 2020).
